# Supplementary material for: Simultaneous CRISPR/Cas9‐mediated editing of cassava eIF4E isoforms nCBP‐1 and nCBP‐2 reduces cassava brown streak disease symptom severity and incidence
Source: Plant Biotechnol J. 2018 Oct 5;17(2):421–34. doi: 10.1111/pbi.12987 (PMC6335076; doi:10.1111/pbi.12987)
Supplement: Supplementary file 1 — Figure S1 Roles of host eIF4E‐potyvirus VPg interaction and sources of recessive resistance. [file PBI-17-421-s001.pdf]

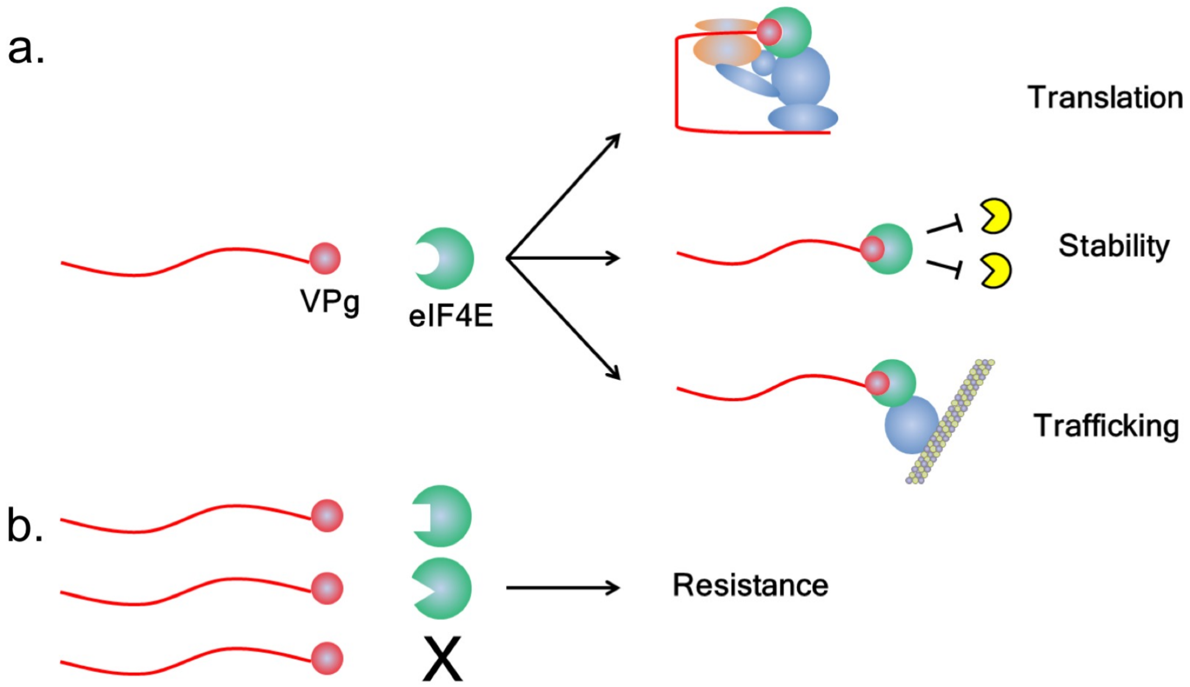

Figure S1. Roles of host eIF4E-potyvirus VPg interaction and sources of recessive resistance.
(a) Linkage of potyvirus VPg to its binding site on eIF4E can provide translation initiation via recruitment of necessary factors and ribosomal subunits, genomic stability via protection from host-encoded exonucleases, and intracellular trafficking via eIF4G microtubule binding activity. (b) Non-conservative amino acid changes and gene deletions that abolish VPg-eIF4E binding removes above described roles, therefore conferring recessive resistance.
