# Supplementary material for: Simultaneous CRISPR/Cas9‐mediated editing of cassava eIF4E isoforms nCBP‐1 and nCBP‐2 reduces cassava brown streak disease symptom severity and incidence
Source: Plant Biotechnol J. 2018 Oct 5;17(2):421–34. doi: 10.1111/pbi.12987 (PMC6335076; doi:10.1111/pbi.12987)
Supplement: Supplementary file 2 — Figure S2 TuMV VPg purified from E. coli can associate with plant eIF(iso)4E in planta. [file PBI-17-421-s014.pdf]

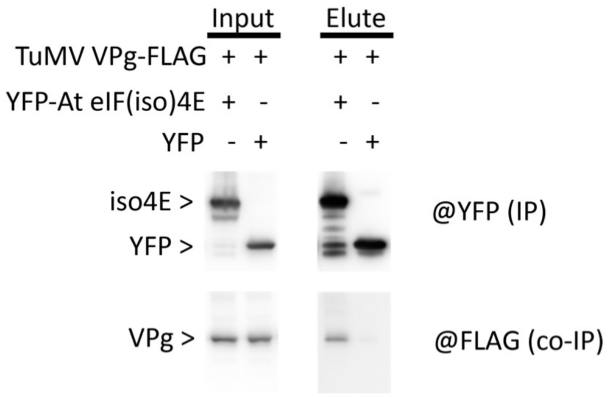

Figure S2. TuMV VPg purified from *E. coli* can associate with plant eIF(iso)4E *in planta*.
Immunoprecipitation of YFP-*Arabidopsis* eIF(iso)4E, but not YFP alone, co-immunoprecipitates TuMV VPg-3xFLAG. YFP and YFP-*Arabidopsis* eIF(iso)4E was co-expressed with *Tomato bushy stunt virus* p19 in *Nicotiana benthamiana* leaves and harvested 48 hours post agroinfiltration. 6xHIS-VPg-6xHIS-3xFLAG was purified from *E. coli* and 6 ug of protein was added to clarified leaf extracts.
