# Supplementary material for: Simultaneous CRISPR/Cas9‐mediated editing of cassava eIF4E isoforms nCBP‐1 and nCBP‐2 reduces cassava brown streak disease symptom severity and incidence
Source: Plant Biotechnol J. 2018 Oct 5;17(2):421–34. doi: 10.1111/pbi.12987 (PMC6335076; doi:10.1111/pbi.12987)
Supplement: Supplementary file 3 — Figure S3 Method for generating CRISPR/Cas9‐mediated gene edited cassava. [file PBI-17-421-s013.pdf]

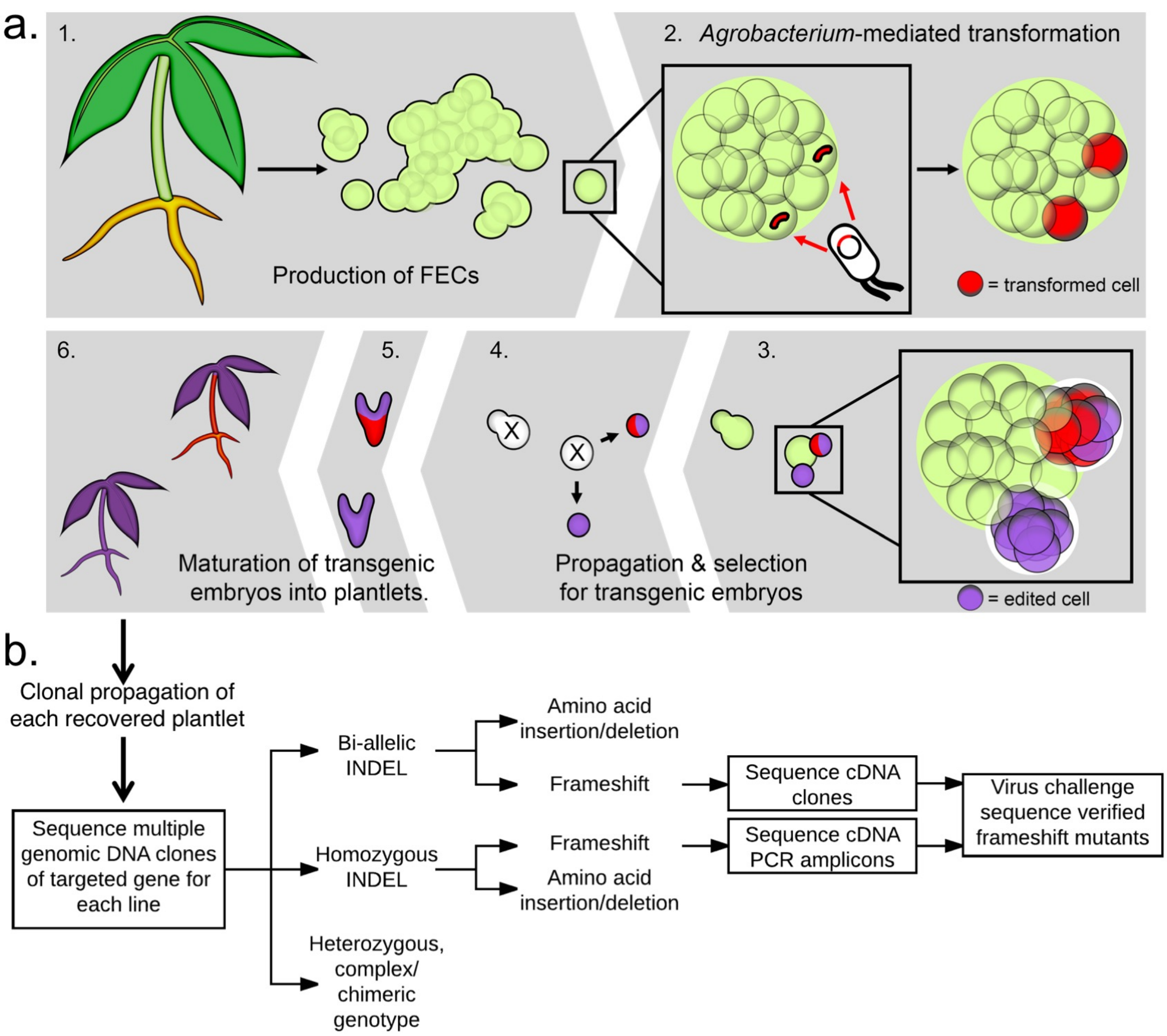

Figure S3. Method for generating CRISPR/Cas9 mediated gene edited cassava

(a) Transgenic cassava are produced via *Agrobacterium*-mediated transformation of friable embryogenic calli (FEC). 1) FEC are induced from somatic tissues by placing the latter on growth media supplemented with picloram. FEC are comprised of aggregated spheroid embryogenic units. Individual units (boxed in panel 1 and enlarged in panel 2) range from a few cells to 1 mm in diameter. 2) FEC are transformed with CRISPR/Cas9 constructs through co-culture with *Agrobacterium tumefaciens.* Red semi-circles denote TDNA fragments and red spheres denote transformed cells. 3) Cells on the surface of embryogenic units, transformed or untransformed, divide to produce new embryogenic units. CRISPR/Cas9 editing can occur prior to or after division. Edited cells are colored purple. 4) Antibiotic selection kills mother and untransformed daughter embryoids. Dead cells marked with “X”. Transformed embryogenic units are spread over selective media and form colonies. One mature embryo per colony is recovered (5), and develops into a plantlet (6). Each regenerated plant is clonally propagated and referred to as a mutant line. (b) Workflow for mutant genotype characterization and line selection.
