# Supplementary material for: Simultaneous CRISPR/Cas9‐mediated editing of cassava eIF4E isoforms nCBP‐1 and nCBP‐2 reduces cassava brown streak disease symptom severity and incidence
Source: Plant Biotechnol J. 2018 Oct 5;17(2):421–34. doi: 10.1111/pbi.12987 (PMC6335076; doi:10.1111/pbi.12987)
Supplement: Supplementary file 4 — Figure S4 CRISPR/Cas9‐induced mutagenesis evident in nCBP‐1 (a) and nCBP‐2 (b) via restriction enzyme site loss (RESL). [file PBI-17-421-s015.pdf]

a.

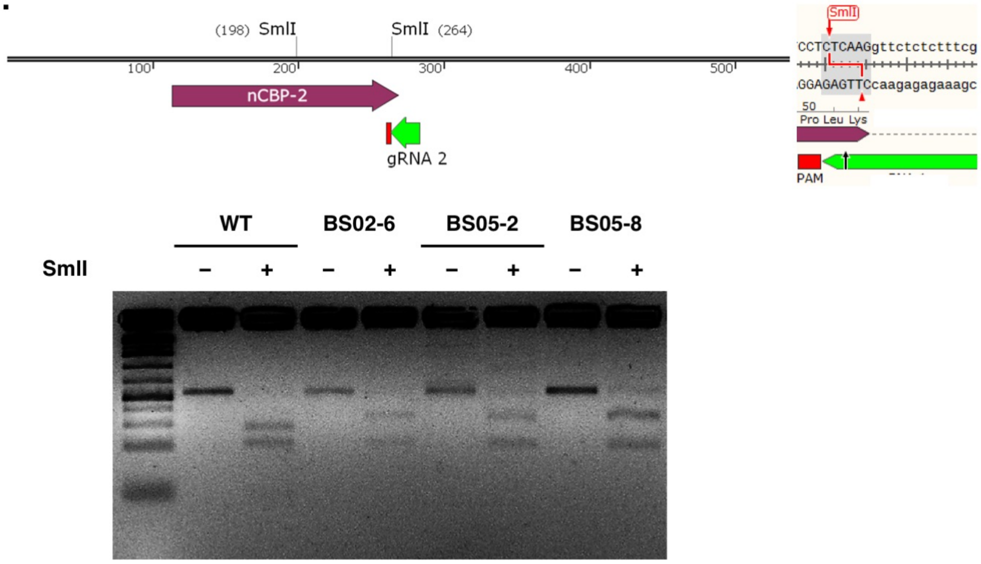

b.

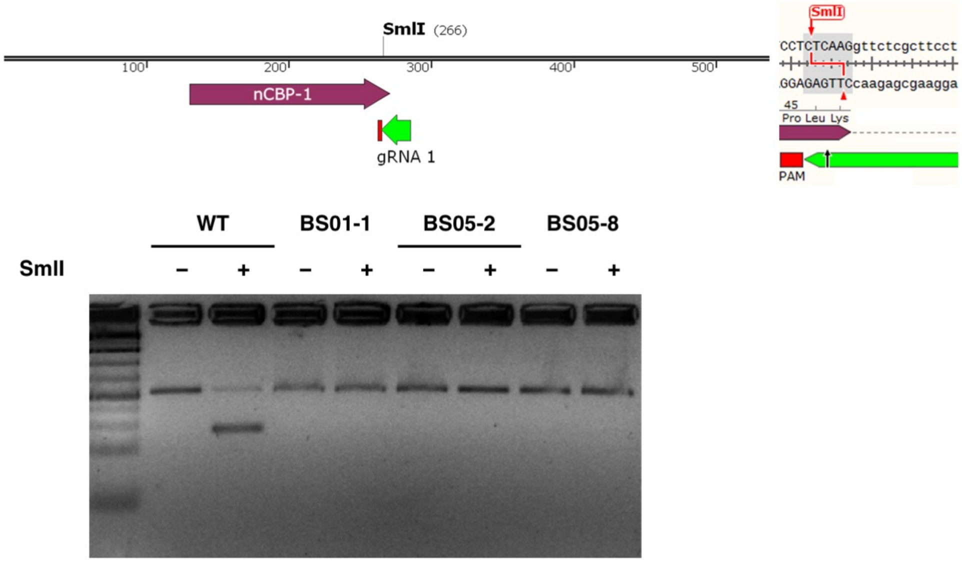

Figure S4. CRISPR-induced mutagenesis evident in nCBP-1 (a) and nCBP-2 (b) via restriction enzyme site loss (RESL). PCR amplicons of targeted regions were digested with SmlI. Map of amplicons with nCBP exon (purple), protospacer adjacent motif (red), gRNA spacer (green), predicted Cas9 cut site (black arrow), and overlapping SmlI restriction enzyme recognition site (bold, red). Bands are measured relative to O'Gene Ruler 1 kb Plus Ladder. Experimental banding pattern is consistent with predicted RESL.
