# Supplementary material for: Simultaneous CRISPR/Cas9‐mediated editing of cassava eIF4E isoforms nCBP‐1 and nCBP‐2 reduces cassava brown streak disease symptom severity and incidence
Source: Plant Biotechnol J. 2018 Oct 5;17(2):421–34. doi: 10.1111/pbi.12987 (PMC6335076; doi:10.1111/pbi.12987)
Supplement: Supplementary file 5 — Figure S5 CRISPR/Cas9–induced mutagenesis creates out‐of‐frame mRNAs. [file PBI-17-421-s016.pdf]

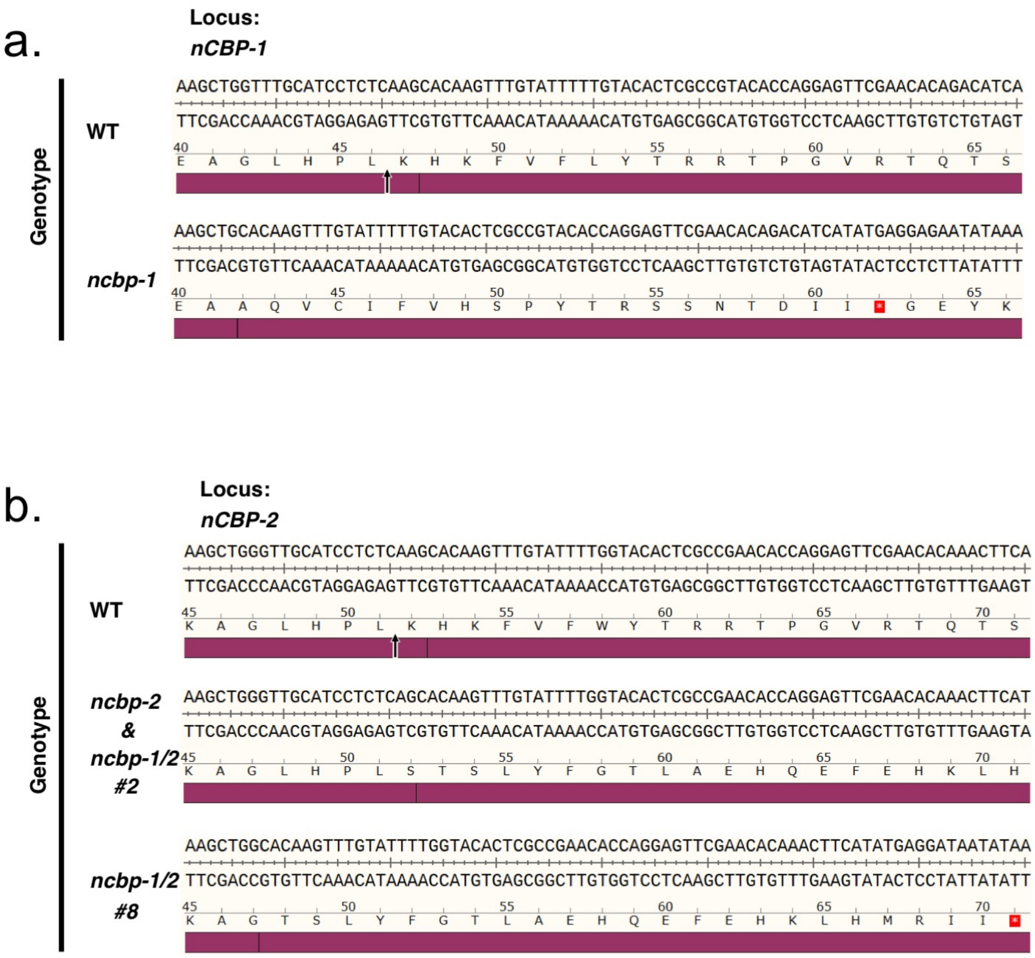

Figure S5. CRISPR/Cas9–induced mutagenesis creates out of frame mRNAs.
Exon 1 and exon 2 splice junction of *nCBP-1* (a) and *nCBP-2* (b) were examined via sequence analysis of cDNA. Frameshifting is observed for all *ncbp* single and double mutants. Predicted Cas9 cut site is shown as a black arrow. STOP codon is shown as starred, red box.
