# Supplementary material for: Simultaneous CRISPR/Cas9‐mediated editing of cassava eIF4E isoforms nCBP‐1 and nCBP‐2 reduces cassava brown streak disease symptom severity and incidence
Source: Plant Biotechnol J. 2018 Oct 5;17(2):421–34. doi: 10.1111/pbi.12987 (PMC6335076; doi:10.1111/pbi.12987)
Supplement: Supplementary file 6 — Figure S6 ncbp‐1 ncbp‐2 double mutants exhibit slowed CBSV symptom onset. [file PBI-17-421-s002.pdf]

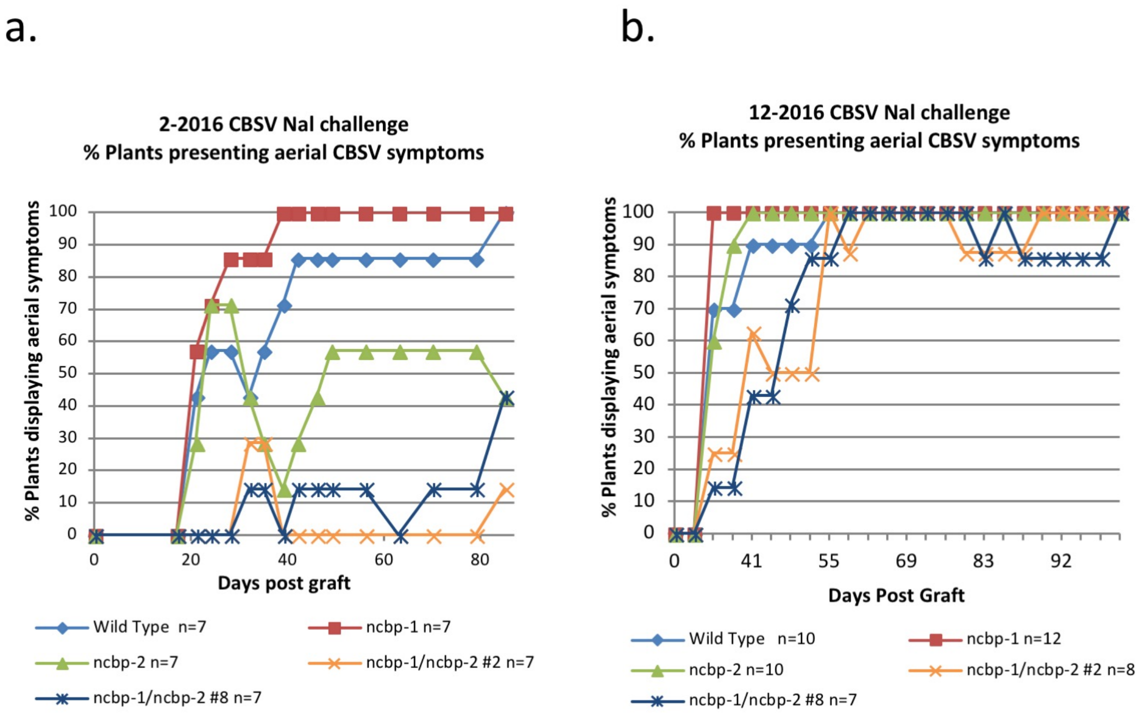

Figure S6. *ncbp-1 ncbp-2* double mutants exhibit slowed CBSV symptom onset.
(a), (b), CBSV aerial symptom incidence for challenges initiated in February and December of 2016, respsectively. *ncbp* double mutants consistently exhibit delayed symptom onset relative to wild-type and single mutants. Incidence is reported as percent of wild type, *ncbp-1*, *ncbp-2*, or *ncbp-1 ncbp-2* plants, bud-graft inoculated with CBSV Naliendele (n≥7), displaying any level of leaf chlorosis or stem streaking.
