# Supplementary material for: Simultaneous CRISPR/Cas9‐mediated editing of cassava eIF4E isoforms nCBP‐1 and nCBP‐2 reduces cassava brown streak disease symptom severity and incidence
Source: Plant Biotechnol J. 2018 Oct 5;17(2):421–34. doi: 10.1111/pbi.12987 (PMC6335076; doi:10.1111/pbi.12987)
Supplement: Supplementary file 7 — Figure S7 ncbp‐1 ncbp‐2 double mutants exhibit reduced aerial CBSV symptom severity. [file PBI-17-421-s003.pdf]

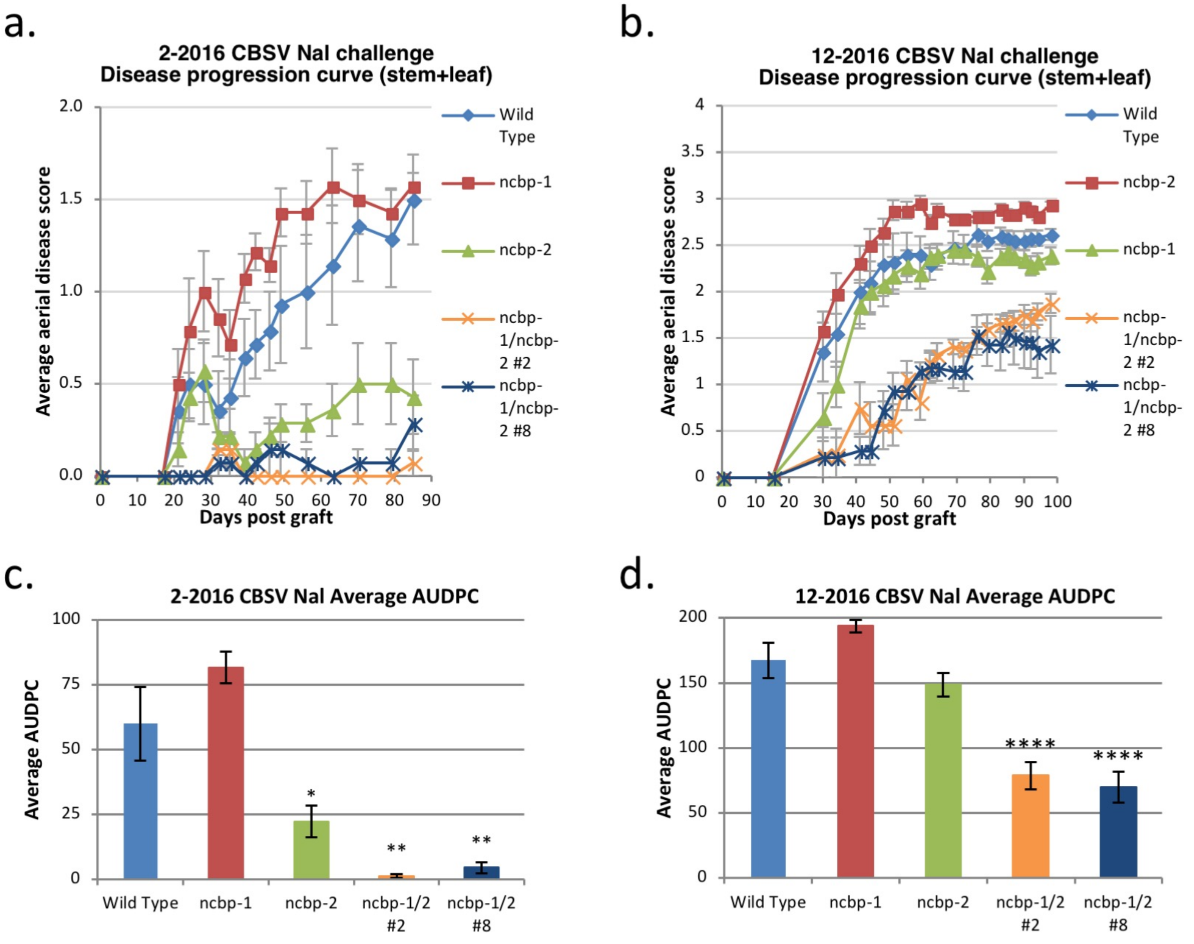

Figure S7. *ncbp-1 ncbp-2* double mutants exhibit reduced aerial CBSV symptom severity.
(a), (b), disease progression curves of wild type, *ncbp-1*, *ncbp-2*, or *ncbp-1 ncbp-2* plants bud-graft inoculated with CBSV Naliendele (n≥7). Leaf and stem symptoms were each scored on a 0-4 scale and averaged to obtain an aerial score. (c), (d), average area under the disease progression curve (AUDPC) derived from data plotted in (a) and (b). Error bars indicate standard error of the mean. Statistical differences were detected by Welch's t-test, α=0.05, *≤0.05, **≤0.01, ****≤0.0001.
