# Supplementary material for: Simultaneous CRISPR/Cas9‐mediated editing of cassava eIF4E isoforms nCBP‐1 and nCBP‐2 reduces cassava brown streak disease symptom severity and incidence
Source: Plant Biotechnol J. 2018 Oct 5;17(2):421–34. doi: 10.1111/pbi.12987 (PMC6335076; doi:10.1111/pbi.12987)
Supplement: Supplementary file 8 — Figure S8 ncbp‐1/ncbp‐2 stem symptom severity is consistently reduced across all experiments. [file PBI-17-421-s004.pdf]

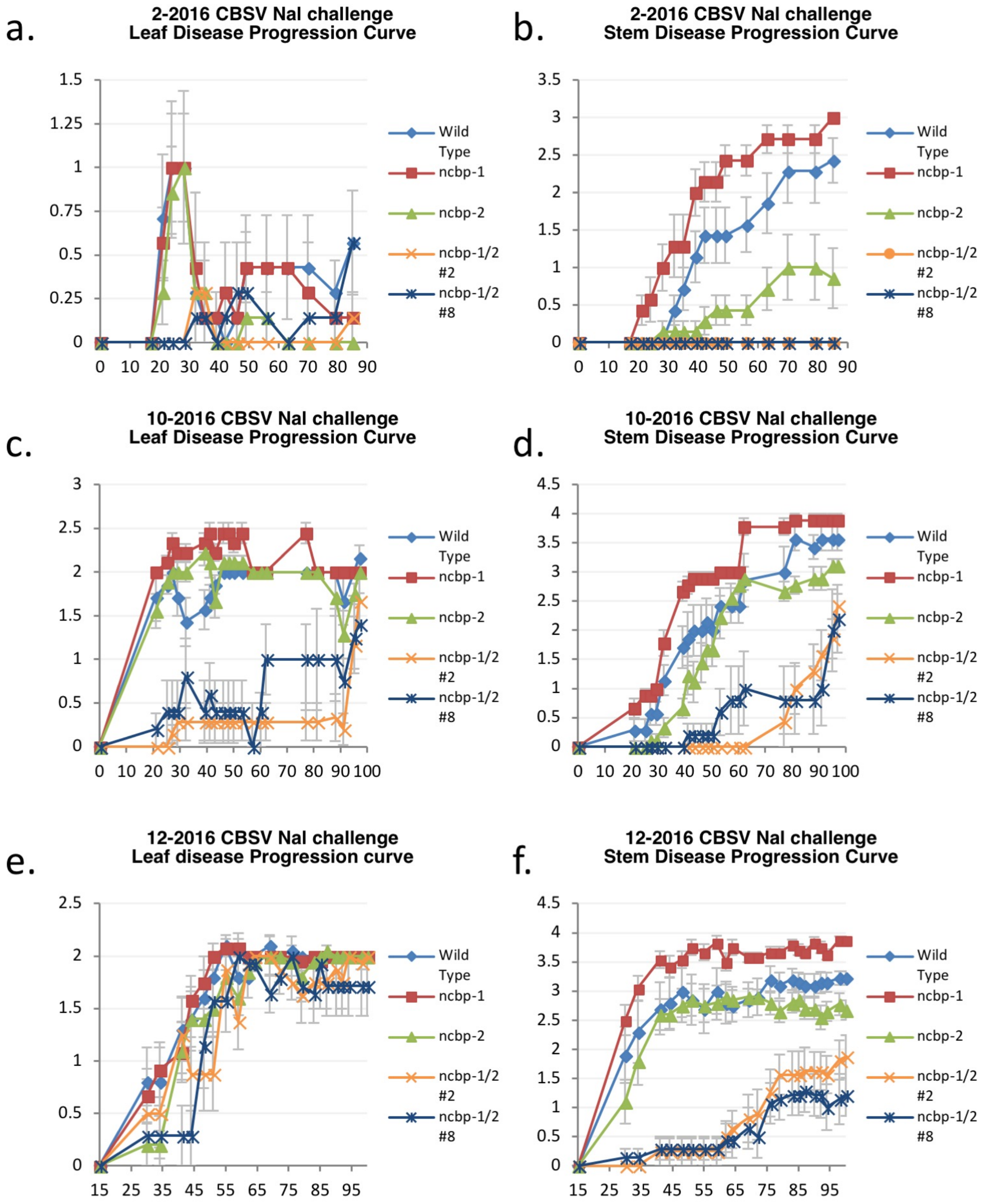

Figure S8. *ncbp-1/ncbp-2* stem symptom severity is consistently reduced across all experiments.
Separate leaf and stem disease progression curves for wild type, *ncbp-1*, *ncbp-2*, or *ncbp-1 ncbp-2* plants bud-graft inoculated with CBSV Naliendele (n≥5). Leaf and stem symptoms were each scored on a 0-4 scale. (a), (c), and (e) represent leaf disease progression curves from three different experiments while (b), (d), and (f) represent corresponding stem disease progression curves. Error bars represent standard error of the mean.
