# Supplementary material for: Simultaneous CRISPR/Cas9‐mediated editing of cassava eIF4E isoforms nCBP‐1 and nCBP‐2 reduces cassava brown streak disease symptom severity and incidence
Source: Plant Biotechnol J. 2018 Oct 5;17(2):421–34. doi: 10.1111/pbi.12987 (PMC6335076; doi:10.1111/pbi.12987)
Supplement: Supplementary file 9 — Figure S9 12‐2016 CBSV challenge leaf symptom severity is similar across all genotypes. [file PBI-17-421-s005.pdf]

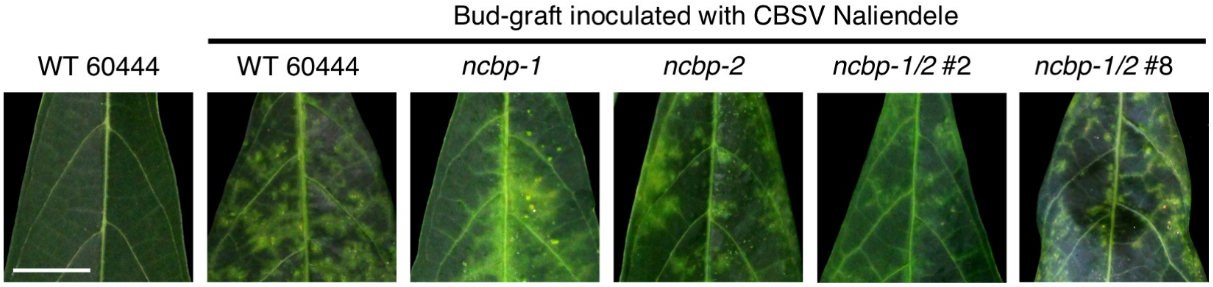

Figure S9. 12-2016 CBSV challenge leaf symptom severity is similar across all genotypes.
Wild type, *ncbp-1*, *ncbp-2*, or *ncbp-1 ncbp-2* plants bud-graft inoculated with CBSV Naliendele isolate all develop widespread chlorotic leaf symptoms. Leaf images were taken near 12-2016 challenge endpoint. Scale bar denotes one centimeter.
