# Supplementary material for: Simultaneous CRISPR/Cas9‐mediated editing of cassava eIF4E isoforms nCBP‐1 and nCBP‐2 reduces cassava brown streak disease symptom severity and incidence
Source: Plant Biotechnol J. 2018 Oct 5;17(2):421–34. doi: 10.1111/pbi.12987 (PMC6335076; doi:10.1111/pbi.12987)
Supplement: Supplementary file 10 — Figure S10 Leaf CBSV‐Naliendele quantitation at challenge endpoint does not reveal consistent differences in foliar virus titre. [file PBI-17-421-s006.pdf]

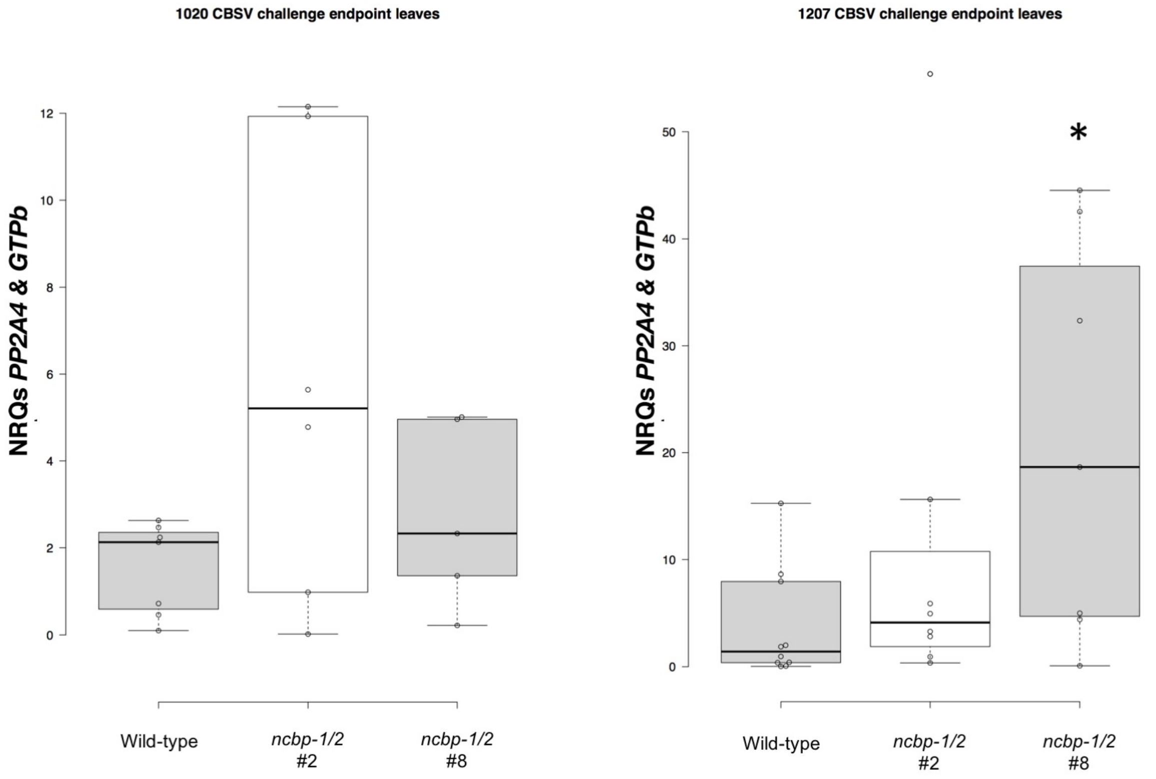

FigureS10. Leaf CBSV Naliendele quantitation at challenge enpdoint does not reveal consistent differences in foliar virus titer. Quantitative real time PCR analysis of endpoint CBSV-Naliendele titer in wild type, *ncbp-1/2* #2, and *ncbp-1/2* #8 leaf tissue. Leaf samples were collected from the first fully expanded leaf. CBSV *HAM1-LIKE* was normalized to *PP2A4* and *GTPb* (*Manes.09G039900* and *Manes.09G086600*). n≥5 per genotype. Whiskers span the interquartile range, solid bars indicate the median of scores. Significant differences were detected with a Mann-Whitney U-test, n≥5, α=0.05, *≤0.05.
