# Supplementary material for: Simultaneous CRISPR/Cas9‐mediated editing of cassava eIF4E isoforms nCBP‐1 and nCBP‐2 reduces cassava brown streak disease symptom severity and incidence
Source: Plant Biotechnol J. 2018 Oct 5;17(2):421–34. doi: 10.1111/pbi.12987 (PMC6335076; doi:10.1111/pbi.12987)
Supplement: Supplementary file 11 — Figure S11 nCBP‐2 is highly expressed in storage roots. [file PBI-17-421-s007.pdf]

Tissue specific expression of cassava *eIF4E* isoforms

Gene	FEC	Fibrous Root	Lateral Bud	Leaf	Mid Vein	OES	Petiole	RAM	SAM	Stem	Storage root
eIF(iso)4E-1	51	28	19	15	12	42	20	70	36	26	28
eIF(iso)4E-2	85	65	49	72	48	81	67	148	74	73	73
nCBP-2	154	185	362	96	113	597	105	142	165	121	1259
nCBP-1	15	51	23	117	91	11	121	38	18	71	114
eIF4E	72	94	74	69	61	72	82	210	104	89	87

shiny.danforthcenter.org/cassava_atlas/

Color Scale

FPKM

210+

210

0

Figure S11. *nCBP-2* is highly expressed in storage roots.
Heat map describing tissue specific expression of cassava eIF4E isoforms. *nCBP-2* is expressed roughly 10 to 45 fold more than other *eIF4E* isoforms in storage roots. Data was extracted from the Bart Lab Cassava Atlas (http://shiny.danforthcenter.org/cassava_atlas/). Expression values are defined as fragments per kilobase of transcript per million mapped reads (FPKM).
