# Supplementary material for: Simultaneous CRISPR/Cas9‐mediated editing of cassava eIF4E isoforms nCBP‐1 and nCBP‐2 reduces cassava brown streak disease symptom severity and incidence
Source: Plant Biotechnol J. 2018 Oct 5;17(2):421–34. doi: 10.1111/pbi.12987 (PMC6335076; doi:10.1111/pbi.12987)
Supplement: Supplementary file 12 — Table S1 Genotypes of all transgenic T0 cassava lines. [file PBI-17-421-s008.docx]

| **Target Gene** | **Construct** | **Line** | **Target Position** | **Mutation Zygosity** | **Genotype** | **Effect** |
| --- | --- | --- | --- | --- | --- | --- |
| nCBP-1 | BS01 | 1 | 133 | Homozygous | d3 | FS |
|  |  | 3 | 133 | Complex | d4, i1, d1, d2 | FS, FS, FS, FS |
|  |  | 4 | 133 | Bi-allelic | d3, d9 | FS, FS |
|  |  | 5 | 133 | WT | WT | No effect |
|  |  | 6 | 133 | Homozygous | i1 | FS |
|  |  | 7 | 133 | Complex | d4, d1, d19, d34i16 | FS, FS, FS, FS |
|  | BS03 | 2 | 2677 | WT | WT | No effect |
|  |  | 3 | 2677 | Homozygous | d9 | FS |
|  |  | 4 | 2677 | Bi-allelic | i3d2, i1 | FS, No effect |
|  |  | 5 | 2677 | Homozygous | i1 | No effect |
|  |  | 6 | 2677 | Homozygous | d1 | FS |
|  |  | 7 | 2677 | Bi-allelic | d3, i1 | 1 AA deleted and 1 AA changed, No effect |
|  |  | 8 | 2677 | Bi-allelic | d12, i1 | FS, No effect |
|  |  | 9 | 2677 | Homozygous | i1 | No effect |
|  |  | 10 | 2677 | Bi-allelic | d93i2, d2 | FS, FS |
|  |  | 11 | 2677 | Bi-allelic | d5, d1 | FS, FS |
| nCBP-2 | BS02 | 1 | 148 | Complex | d3, d6, d7, i2d8 | 1 AA deletion and 1 AA changed, FS, FS, FS |
|  |  | 2 | 148 | Bi-allelic | d5, d2 | FS, FS |
|  |  | 4 | 148 | Bi-allelic | i4d127, i8d20 | FS, FS |
|  |  | 5 | 148 | Complex | d17, d52, d1, i2d4 | FS, FS, FS, FS |
|  |  | 6 | 148 | Homozygous | d1 | FS |
|  |  | 7 | 148 | Bi-allelic | d1, d17 | FS, FS |
|  | BS04 | 1 | 2763 | Heterozygous | WT, i1 | No effect, FS |
|  |  | 2 | 2763 | Bi-allelic | i1, d7 | FS, FS |
|  |  | 3 | 2763 | Complex | i1, d7, d4 | FS, FS, FS |
|  |  | 4 | 2763 | Bi-allelic | d4, i1 | FS, FS |
|  |  | 5 | 2763 | Bi-allelic | d2, d6 | FS, 2 AA deletion |
|  |  | 6 | 2763 | WT | WT | No effect |
|  |  | 8 | 2763 | Homozygous | i1 | FS |
|  |  | 9 | 2763 | Bi-allelic | d2, d7 | FS, FS |
|  |  | 10 | 2763 | Bi-allelic | i1d8, d2 | FS, FS |
|  |  | 11 | 2763 | Bi-allelic | d2, i1d10 | FS, FS |
|  |  | 12 | 2763 | Bi-allelic | d5, d11 | FS, FS |
|  |  | 13 | 2763 | Homozygous | d2 | FS |
|  |  | 14 | 2763 | Bi-allelic | d7, d2 | FS, FS |
|  |  | 15 | 2763 | Bi-allelic | i1, d2 | FS, FS |
|  |  | 16 | 2763 | Bi-allelic | i1, d5 | FS, FS |
| nCBP-1/2 | BS05 | 1 | 148 | Bi-allelic | d7, d1 | FS, FS |
|  |  |  | 133 | Bi-allelic | d2, d4 | FS, FS |
|  |  | 2 | 148 | Homozygous | d11 | FS |
|  |  |  | 133 | Homozygous | i1 | FS |
|  |  | 3 | 148 | Bi-allelic | i1, d1 | FS, FS |
|  |  |  | 133 | Bi-allelic | d1, d5 | FS, FS |
|  |  | 4 | 148 | Bi-allelic | d5, d4 | FS, FS |
|  |  |  | 133 | Bi-allelic | d2, d1 | FS, FS |
|  |  | 5 | 148 | Bi-allelic | i1, d6 | FS, FS |
|  |  |  | 133 | Bi-allelic | d4, d9 | FS, FS |
|  |  | 6 | 148 | WT | WT | No effect |
|  |  |  | 133 | WT | WT | No effect |
|  |  | 7 | 148 | Bi-allelic | i1, d15 | FS, FS |
|  |  |  | 133 | Bi-allelic | d3, d5 | FS, FS |
|  |  | 8 | 148 | Homozygous | d1 | FS |
|  |  |  | 133 | Bi-allelic | d3, d1 | FS, FS |

Table S1. Genotypes of all transgenic T_0_ cassava lines.

WT, wild-type alleles; bi-allelic, two different mutated alleles; heterozygous, wild-type and mutated alleles; complex, more than two mutated alleles. d# and i# refer to deletions and insertions, respectively, with the number of bases mutated denoted by #. Highlighted transgenic events were used in CBSV/UCBSV challenge assays. FS, Frameshift
