# Supplementary figures and images for: Simultaneous CRISPR/Cas9‐mediated editing of cassava eIF4E isoforms nCBP‐1 and nCBP‐2 reduces cassava brown streak disease symptom severity and incidence
Source: Plant Biotechnol J. 2018 Oct 5;17(2):421–34. doi: 10.1111/pbi.12987 (PMC6335076; doi:10.1111/pbi.12987)

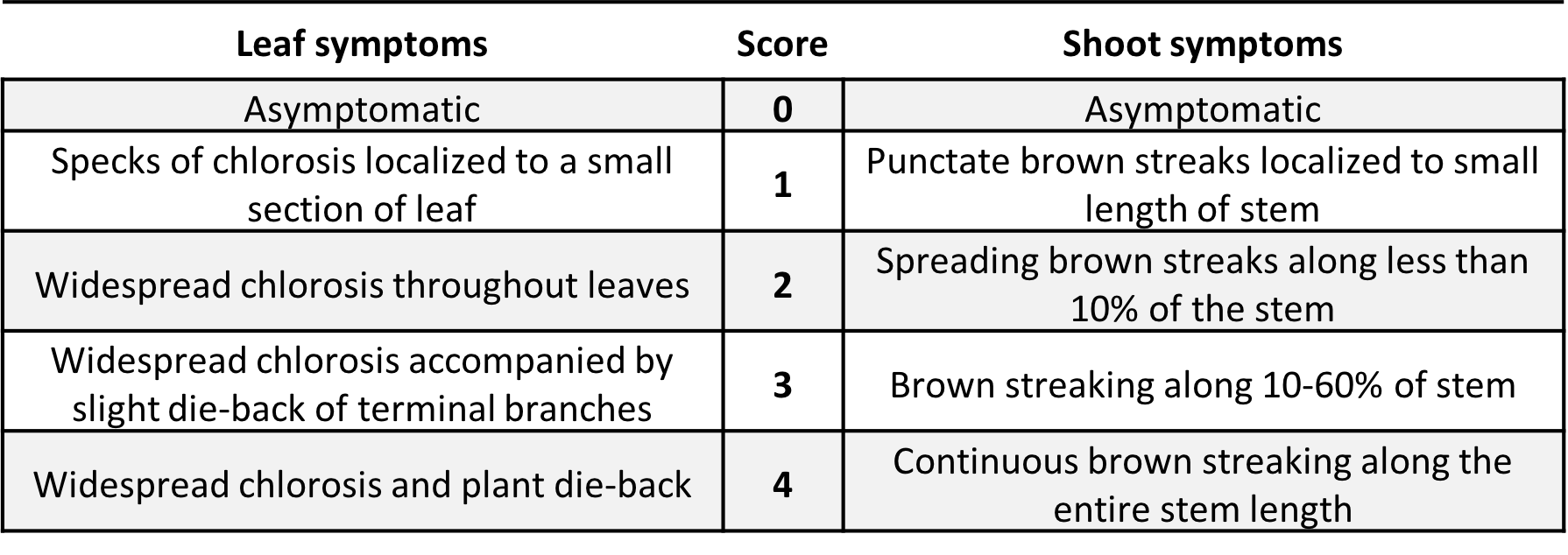
Table S4. Aerial symptom scoring scale.

Supplement: Supplementary file 15 — Table S4 Aerial symptom scoring scale. [file PBI-17-421-s011.docx]
